# The estimated incidence of pertussis in people aged 50 years old in the United States, 2006–2010

**DOI:** 10.1186/s12879-015-1269-1

**Published:** 2015-11-19

**Authors:** Cristina Masseria, Girishanthy Krishnarajah

**Affiliations:** Global Health & Value, Pfizer Inc., 235 E 42nd street, New York, NY 10017 USA; Vaccines, GlaxoSmithKline Vaccines, 5 Crescent Dr, Philadelphia, PA 19112 USA

**Keywords:** Pertussis, Incidence, Whooping cough, Underreporting, Adults

## Abstract

**Background:**

Pertussis is believed to be widely underreported and under-recognized, particularly among adults. The aim of this study was to estimate the incidence of private practitioner-attended cough illness that could be attributed to *Bordetella pertussis* in adults aged ≥50 years in the US.

**Methods:**

Multiple linear regressions were employed to estimate the overall incidence of pertussis. Data were extracted from IMS’ private practice database of longitudinal, patient-level claims and IMS’ commercial laboratory database during 4/1/2006–12/31/2010. Patients were ≥50 years old and had ≥1 ICD-9-CM claim for cough illness relating to pertussis, cough, or acute bronchitis. Pertussis positive laboratory tests, seasonal and secular variables were used for estimating the *B. pertussis* attributable fraction of cough illness*.*

**Results:**

During the study period, there were 20.7 million cases of cough illness among people aged 50–64 and 27.5 million cases among those ≥65; of which the model attributed 2.5 and 1.7 %, respectively, to *B. pertussis*. The estimated incidences of cough illness attributed to *B. pertussis* during the study period were on average 202 and 257/100,000 among people aged 50–64 and ≥65 years, respectively, and increased over the years in both age groups. Depending on the year, estimated pertussis incidences were 42 to 105 times higher than medically attended ones in the same database.

**Conclusions:**

These findings indicate that the *B. pertussis* disease incidence in adults aged ≥50 years is significantly higher than generally estimated. Additional research regarding pertussis reporting and diagnosis in the adult populations is needed to validate these findings.

## Background

Pertussis (whooping cough), caused by *Bordetella pertussis (B. pertussis)*, is a highly contagious respiratory tract illness [1]. Although introduction of universal infant pertussis immunization led to an initial reduction in incidence [2, 3], this has recently increased again worldwide, potentially due to factors such as waning immunity, better diagnostic testing, active surveillance, altered vaccine characteristics, and increased awareness [2, 4]. Among adults, pertussis generally results in a prolonged cough, and can cause pharyngeal discomfort, influenza-like symptoms, hoarseness, sinus pain, and may lead to complications such as urinary incontinence, rib fracture, pneumothorax, inguinal hernia, and otitis media [5]. In addition, adults can pass infection to young infants [6, 7], in whom severe complications and death can occur [6, 8].

In the United States, the Advisory Committee on Immunization Practices (ACIP) has consistently advised that vaccination offers the best protection against pertussis [9]. In 2006 ACIP recommended the administration of a reduced antigen content tetanus toxoid, diphtheria toxoid, and acellular pertussis (Tdap) vaccine to all adolescents and adults 19 through 64 years of age [3]. In February 2012, the ACIP extended the Tdap recommendation to adults aged 65 years and older [10].

There is a paucity of information on the true incidence of pertussis, as many cases are not reported and/or laboratory confirmed [11–14]. Underreporting and under diagnosis is considered to be a major issue, particularly among adults. In addition, adults tend to seek medical care much later in the course of the illness, when confirmation of the disease by laboratory testing is less sensitive. Distinguishing pertussis from other respiratory illnesses is challenging and clinicians are not often aware of pertussis among adults. Confirmation of disease via culture and polymerase chain reaction (PCR) also poses challenges among adults [11–14].

A better understanding of pertussis incidence in mature adults (those >50 years) is necessary to develop effective clinical approaches and public health programs to curb its spread. In absence of direct estimates of disease incidence, inferential statistical methods can be used to estimate the true disease incidence of pertussis infections. This methodology has been used extensively to estimate the incidence of other underreported diseases that are rarely confirmed by laboratory testing, such as influenza [15–17]. The covariation of etiology-specific cough illness and laboratory-confirmed *B. pertussis* infections throughout the length of the time series is used for attributing a proportion of cough illness to pertussis infections, after correcting for seasonal and secular trends. The objective of this study was to estimate the incidence of private practitioner-attended cough illness that could be statistically attributed to *B. pertussis* in adults aged ≥50 years in the US. The data presented here was part of a larger study. Phase I results have recently been published [18] and will be used for comparing the estimated pertussis incidence obtained with this model exercise with medically attended incidence.

## Methods

### Data sources

Data were extracted from IMS’ private practice database (CMS-1500) of longitudinal, patient-level claims (April 2006 to December 2010). This private practitioner claims database contains claims completed for insured patients seen by private practitioners across the 50 states of America. This database constitutes the largest ongoing tracking program of outpatient office visit data in the US, with approximately 1 billion entries and over 80 million claims per year submitted by more than 870,000 physicians per month. Between 2006 and 2010 over 100 million unique patients aged 50 and over were observed in the database.

Data regarding *B. pertussis* laboratory tests were extracted from IMS’ commercial laboratory partner (April 2006 to December 2010), which covered approximately 40 % of US-performed testing. Laboratory tests used for *B pertussis* were: Polymerase Chain Reaction; PCP antigen detection, culture or singe sera IgA, IgG, and or IgM titers.

Data were de-identified by assigning each patient with a longitudinal identifier. The databases are third-party certified as being compliant with the Health Insurance Portability and Accountability Act.

### Sample

Patients were included if they were ≥50 years old, with a date of service between 4/1/2006 and 12/31/2010 and with a cough illness (Table 1). More information on the demographic and baseline characteristics of patients included in this study is reported in the Phase I study [18].

**Table 1:** PCSA population: projected rates of cough illness/ ICD-9 pertussis and actual laboratory tests

Year	PCSA population		Cough illness^a^		ICD-9 pertussis^b^		Incidence of medically reported pertussis per 100,000^c^		Lab tests total (% positive)	
	50-64 y	≥65 y	50-64 y	≥65 y	50-64 y	≥65 y	50-64 y	≥65 y	50-64	≥65 y
2006^d^	47,692,685	34,408,408	2,420,259	3,410,356	1580	1064	3.0	2.9	2694 (36.2 %)	1415 (29.1 %)
2007	49,339,902	34,949,825	3,631,552	4,954,117	1332	1020	2.5	2.7	4121 (33.3 %)	2007 (27.9 %)
2008	50,995,966	35,448,456	4,422,844	5,981,570	1176	1285	2.1	3.3	4382 (37.6 %)	2103 (29.7 %)
2009	52,378,740	36,335,561	4,980,846	6,388,537	1957	1209	3.5	3.1	4832 (38.7 %)	2631 (35.4 %)
2010	53,604,318	37,372,356	5,256,210	6,763,574	2719	1790	4.6	4.4	6621 (38.2 %)	4141 (36.2 %)
Total	254,011,611	178,514,606	20,711,712	27,498,154	8764	6368	3.15	3.27	22,650 (37.1 %)	12,297 (32.8 %)

*PCSA* Primary Census Statistical Area

^a^ICD-9 codes: 033.0 (pertussis/whooping cough due to *B. pertussis*), 033.9 (pertussis/whooping cough due to unspecified organism), 484.3 (pneumonia in whooping cough), 786.2 (cough), 466.0 (acute bronchitis)

^b^ICD-9 codes: 033.0, 033.9, 484.3

^c^For more information on the incidence calculation and the descriptive analysis of the database please refer to the McGuiness et al. (2013) publication [22]

^d^Data were only collected for 9 months during 2006 (April to December)

Pertussis was defined using the following International Classification of Diseases, Ninth Revision (ICD-9) diagnosis codes: pertussis/whooping cough (033.0 [due to *B. pertussis*], 033.9 [unspecified organism], 484.3 [pneumonia in whooping cough]). The definition of cough illness included the above pertussis-related ICD9 codes, and ‘pertussis like conditions’ [cough (786.2), or acute bronchitis (466.0)]. Cough and acute bronchitis (categorized as part of a larger lower-respiratory tract infection group) were chosen because they were the conditions (based on ICD9 codes) most frequently diagnosed in the three months preceding a pertussis diagnosis among patients aged 50 and older in the IMS database [18]. The majority of patients had a ‘pertussis-like condition’ diagnosed in the three months preceding pertussis diagnosis and approximately 90 % of these were acute respiratory conditions, and 43 % had specifically either a cough and/or acute bronchitis diagnosis.

Only patients with positive laboratory tests were included for model estimates.

Data were stratified by age of the patients into 2 groups (50–64, ≥ 65) and aggregated by calendar month.

### Regulatory and ethical considerations

The study was conducted in accordance with the applicable regulatory requirements, the International Conference on Harmonization, Guideline for Good Clinical Practice, subject privacy requirements, and the guiding principles of the Declaration of Helsinki. Data transmitted to the study investigators was clean of all personally identifiable information. No data that could link back to individual patients were included. All data were aggregated prior to sharing with GSK.

### Analysis – estimation of the catchment population/denominator

Incidence rates per 100,000 persons were calculated by dividing the projected number of cough illness and whooping cough cases attributable to *B. pertussis* by the US Census populations [19] according to age group and year and multiplying the product by 100,000. IMS’ private practice database does not capture data from every US private practice, therefore to ensure that the data represent appropriately the US population, the data were projected up to Primary Census Statistical Areas (PCSAs). The total of office-based American Medical Association practitioners [20] was divided by the corresponding number of practitioners in the IMS sample. This was done using IMS’ medical data projection weights [18, 21], estimated by month and physician specialty strata.

Only private practitioners with relevant longitudinal submission of claims were included in the sample to ensure accuracy of the weighted incidence calculations. They represented 9 % (in 2006) to 17 % (in 2010) of all General Practice, Internal Medicine or Geriatrics practitioners in the US [18].

### Analysis – modeling

The model selected for estimating the incidence of cough illness attributable to pertussis has previously been used in an international study of influenza and was chosen because it allows to capture underreporting [15]. Similar models have been used in several therapeutic areas (rotavirus, respiratory syncytial virus, and influenza) [17, 22, 23].

The multiple linear regression model applied to the data to determine the incidence of cough illness that could be attributed to *B. pertussis* is described below:

$$ \mathrm{Cough}\ \mathrm{illness}\ \mathrm{rate} = {\upbeta}_0 + {\upbeta}_1*B.\  pertussis + {\upbeta}_2*\mathrm{sine}\left(2*\mathrm{t}*\uppi /12\right) + {\upbeta}_3*\mathrm{cosine}\left(2*\mathrm{t}*\uppi /12\right) + {\upbeta}_4*\mathrm{t} + {\upbeta}_5*\left(\mathrm{t}*\mathrm{t}\right) + \mathrm{e} $$

The dependent variable, *cough illness rate*, was equal to the cough illness incidence. The term β_0_ was the constant term. *B. pertussis* represented the laboratory tests positive for *B. pertussis* for each month in the time period of interest. β_1_ was the regression coefficient used for estimating the number of cough events attributable to *B. pertussis*. The next two independent variables in the equation and their corresponding coefficients (β_2_ and β_3_) were included to account for seasonal changes in the dependent variable [24–26]. The last two independent variables in the equation and their associated coefficients (β_4_ and β_5_) accounted for linear and non-linear trends in the dependent variable that were not associated with the cough illness/*B. pertussis* positive laboratory test signal in the time series. The variable t represented the month in the time series analysis and *e* represented the error term. Independent variables with non-statistically significant (i.e. p >0.1) coefficients were excluded from the model (the only case was Tdap vaccination). The regressions were run using Stata SE 9 and SAS 9.2.

### Model fit and attribution of cough to *B. pertussis*

The monthly attribution of the cough illness rate to *B. pertussis* was generated by computing the products of the regression coefficient associated with *B. pertussis* (i.e. β_1_) and the monthly count of laboratory tests positive for *B. pertussis*. The annual attribution was obtained by summing the monthly totals within each year. Then, the percentage attribution was calculated using the predicted and observed sums of cough illness in each year as the denominators.

The model-predicted and observed monthly cough illness rates to *B. pertussis* were compared for both age groups, with a satisfactory visual fit of the model.

### Study objective

The objective was to estimate the incidence of private practitioner-attended cough illness that could be attributed to *B. pertussis* in adults aged 50–64 and ≥65 years old in the US.

## Results

### Sample

The total number of patients in the PCSA population, along with the projected numbers of patients with cough illness, the total number of medically attended pertussis (ICD-9) cases, and laboratory tests are reported in Table 1.

### Laboratory diagnosis of pertussis

The number of laboratory tests performed increased over the years (Table 1). During the study period, 8398 laboratory tests were positive for *B. pertussis* among individuals aged 50–64 years corresponding to 37 % of the total tests performed, and 4029 among those aged ≥65 years corresponding to 33 % of total tests performed. Only 45 % of the patients with a positive laboratory test had a pertussis related diagnosis between 14 days prior and 14 days after the index date. There was no clear time trend in the number of positive tests for people 50–64 while for those over 65 years the percentage of positive tests increased over time.

### Pertussis incidence by ICD-9 codes

Between April 2006 and December 2010, 8763 pertussis cases were diagnosed in outpatient settings (ICD9: 033.0, 033.9 and 484.3) among people aged 50–64 and 6369 among people 65 and older (Table 1). The incidence was estimated to vary between 2.1 and 4.6 cases per 100,000 people across the two age groups (50–64 and ≥65; Table 1) [18]. Pertussis infections rose particularly in 2010; the diagnosed incidence was 4.6 and 4.4 per 100,000 among people aged 50–64 and ≥65 respectively.

### Incidence of cough illness

**Fig. 1:**
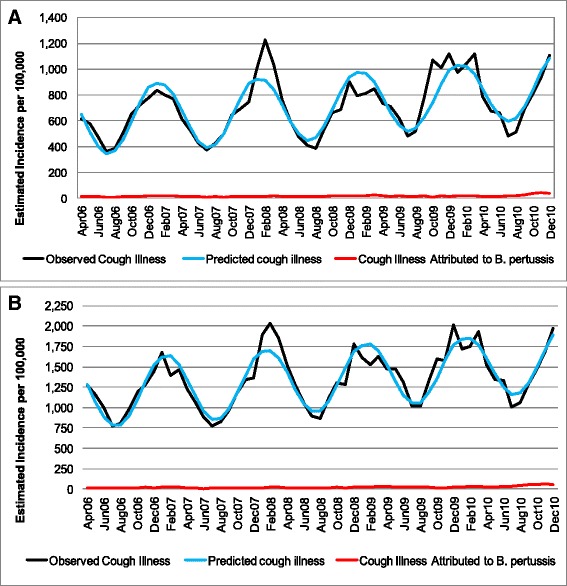
Observed and predicted cough illness by age groups (**a**-50-64; **b** ≥65)

Nearly 21,000,000 and 27,500,000 cases of cough illness were reported among people aged 50–64 and ≥65 years, respectively during the study period (Table 1). Cough illness reporting varied seasonally, ranging from 780 to 2000 per 100,000 depending on the time of the year (Fig. 1a and b), with comparable rates across the years.

### Incidence of cough illness attributable to *B. pertussis*

**Fig. 2:**
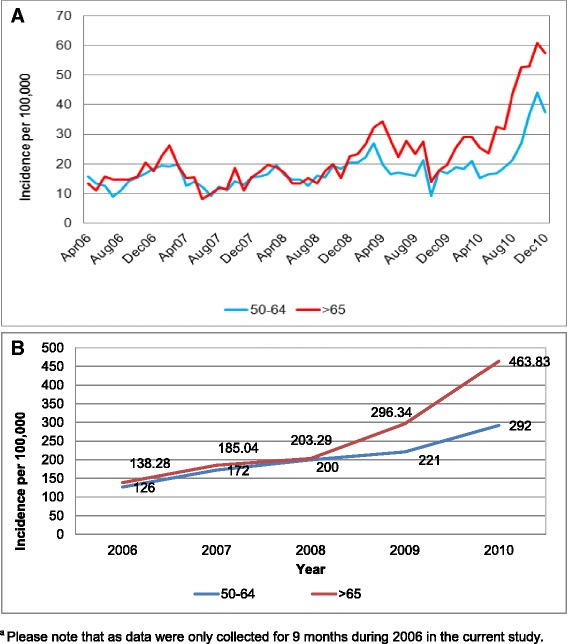
Incidence* of cough illness attributed to *B. pertussis* by month (**a**) and by year (**b**). *Please note that as data were only collected for 9 months during 2006 in the current study, an annual figure was approximated by multiplying by 12/9

The majority of cough illness was unattributed by the model (Fig. 1a and b). The multiple linear regression model attributed 519,078 and 465,337 cases of cough illness to *B. pertussis* infections among people aged 50–64 and ≥65 years, respectively, accounting for 2.5 and 1.7 % of all cough illness, respectively. No clear seasonal variation can be detected (Fig. 2a and b). The annual incidence of cough illness attributed to *B. pertussis* increased over the years with a sharp increase in 2009 and 2010 in particular for the older age group (Fig. 2b). Incidence rates in 2006 were 126 and 138 per 100,000 for people aged 50–64 and ≥65 respectively; by 2008 they increased to 200 and 203 respectively, to reach the value of 292 and 464 in 2010.

Depending on the year, these estimated pertussis incidences were 42 to 105 times higher than the ones reported previously obtained by using only private practitioners diagnosed pertussis/whooping cough case (ICD9 033.0, 033.9 and 484.3) (Table 1).

## Discussion

To the best of our knowledge, this study is the first attempt to quantify the incidence of cough illness attributed to *B. pertussis* via regression modeling among mature adults in the US. The results of our analysis show that while cough is a seasonal disease (Fig. 1a and b) with peaks during the winter period, no clear seasonal trend was observed for estimated pertussis (Fig. 2a). The model attributed approximately 2 % of cough illness to pertussis, with the remaining episodes of cough illness caused by other health conditions. The literature shows that for adolescents and adults *B. pertussis* rates among people with prolonged cough varied from 1 to 17 % [27] when a stringent definition of positive laboratory test is used (antibody response to PT). Ward estimated that *B. pertussis* accounted for 0.7 to 5.7 episodes of cough illness, depending of the duration of cough [28]. Specifically among individuals aged ≥65 reported, between 25 and 50 % of pertussis cases had preceding cough diagnosis [29]. The lower rate of *B. pertussis* among cough illness episodes estimated here can be the results of the inclusion of acute bronchitis and of the inability of differentiating prolonged cough.

The data presented here show that the number of pertussis cases increased from 8764 diagnosed cases (ICD-9: 033.0, 033.9, 484.3) during the study period to approximately 520,000 estimated cases for people aged 50–64 years, and from 6369 to approximately 465,000 for those ≥65. Depending on the year and the age group, incidence rates were 42 to 105 times higher than the ones calculated using only medically diagnosed pertussis cases [18]. In 2010, the estimated incidence was 94 and 264 times higher than national reported incidences (NNDSS) for individuals aged 40–65 and ≥65 years, respectively [30]. Moreover, incidence rates for cough illness attributable to pertussis increased each year, with more than a two folds increase among people ≥65.

Our results are in line with previous US studies on pertussis performed during the mid-to-late 1990s reporting annual rates varying from 176 to 1500/100,000 [28, 31, 32]. The Nanning et al. study, which enrolled 307 adults (≥18 years) into a single-center prospective study [31] estimated a pertussis incidence of 176/100,000 person-years during 1997–2000. The Strebel et al. prospective study enrolled 212 patients aged 10–49 years old with acute paroxysmal cough or cough illness for 7–34 days in Minnesota [32]. On the basis of any positive laboratory result, the estimated annual incidence of pertussis was 507/100,000 person-years. A multicenter prospective study of 2781 healthy subjects, of which 1391 received acellular pertussis vaccine, aged 15–65 years enrolled in a randomized, double-blind trial of an acellular pertussis vaccine reported an annual pertussis incidence of 370–450 cases/100,000 [28]. Hodder et al., specifically analyzed rates of B pertussis among individuals aged 65 and older [29]. Depending on the definition of infection, incidence varied from 19.7 to 3.3 per 100 person-years.

These variations in *B. pertussis* incidence estimations are associated with the specific populations studied as well as the pertussis definitions used.

The results of our study, along with the results of previous US studies, support the claim that pertussis is widely underreported, especially among adults, in whom clinical presentation varies widely [7, 11] . Pertussis is under recognized as a cause of cough, clinical suspicion may be low, and the disease may be mild enough that medical care is not sought. Direct assessment of pertussis disease incidence is not possible because not all cases of pertussis are diagnosed or laboratory confirmed, partly due to laboratory testing being uncommon outside of outbreaks. A call for broader laboratory testing among elderly adults may be considered given the results of this study. Some tests are most sensitive in the early stages of the disease [13, 33], before the adult patient seeks medical attention. Testing at the later stages of the disease results in a higher false negative rate [13, 33] and an underestimation of disease incidence. Improvements in laboratory diagnosis may be associated with a rise in the number of pertussis reported cases. Nevertheless, care must be taken because *B. holmesii* and *B. pertussis* genomes share the same IS481insertion sequence and routine polymerase chain reaction (PCR) techniques can falsely attribute respiratory infection due to *B. holmesii* to *B. pertussis*, ie false positives [34]. Other possible explanations for the rising pertussis incidence are: greater awareness of pertussis; the use of acellular versus whole-cell vaccines; genetic changes in *B. pertussis*; and waning of vaccine-induced immunity [4, 27]. The number of outbreaks of pertussis has increased in the last years [35–38], with obvious health and economic consequences.

The data presented here suggest that *B. pertussis* infection is common in mature adults and particularly among the people aged ≥65. According to the literature, infections in adults are endemic and not cyclical as in children [27]. Vaccination among children and young adults may have shifted the incidence of pertussis to older adults [39]. Because not all adults who get pertussis will experience a severe cough, adults often unknowingly spread pertussis to infants, who are at risk for a severe, sometimes life-threatening illness. These results support the ACIP decision to extend Tdap vaccination to individuals aged 65 and older [10].

### Strengths and limitations

Strengths of the study include the large sample size and the geographical representativeness of the sample. However, the results of this study should be interpreted in the context of its limitations. Claims data are inherently limiting, because they are collected for billing and reimbursement purposes rather than for research. Furthermore, data entry errors at the site of care cannot be corrected.

Only patients seeking medical attention from private practitioners were included. Patients visiting emergency departments and/or hospitals as well as those who do not seek medical attention are not included in our estimates. Moreover, we included only individuals with a whooping cough diagnosis or a pertussis like diagnosis defined as wither cough illness (length of cough illness was not taken into consideration) or acute bronchitis. Therefore, given the conservative definition of pertussis like diagnosis our data likely underestimate the true incidence of pertussis. However, we did include cases of whooping cough due to unspecified organisms, some of which may not have been due to *B. pertussis*, which probably led to a small overestimation in this regard. We did not test whether the results of the analysis may change with a more restrictive definition of pertussis or cough illness.

Measurement error within the current study is also likely. By including only positive *B. pertussis* laboratory tests we may have underestimated the real incidence of pertussis. The retrospective, observational nature of the study should also be considered when interpreting the results. Retrospective analyses demonstrate associations but do not indicate causality and could make the results subject to selection bias.

Lastly, the incidence of cough attributed to *B. pertussis* was based on mathematical modeling. Models try to mimic the reality and are subject to numerous limitations and assumptions. Although we used methodology from the published literature [15, 17, 22, 23], there may be more efficient way of estimated the overall incidence of pertussis.

## Conclusions

Overall, our findings indicate that *B. pertussis* incidence is substantial in adults aged ≥50 years that considerably exceeds the generally reported disease incidence. Although only approximately 2 % of cough illness was attributed to *B. pertussis*, the gross disease burden associated with pertussis is sizeable from a public health perspective. These results highlight the importance of improving clinical awareness of pertussis in adults, as well as the need for better prevention and control of pertussis in adults. Incidence rates of cough illness attributed to *B. pertussis* increased from 2006 to 2010. Further research is needed to better understand the incidence of disease among people over 50 and to validate these findings.

## Abbreviations

ACIP: Advisory Committee on Immunization Practices
DNA: deoxyribonucleic acid
HIPAA: Health Insurance Portability and Accountability Act
ICD-9: International Classification of Diseases, Ninth Revision
Ig: immunoglobulin
PCR: polymerase chain reaction
PCSAs: Primary Census Statistical Areas
Tdap: reduced antigen content tetanus toxoid, diphtheria toxoid, and acellular pertussis

## References

[1] Munoz FM, Keitel WA (2003). Progress in the diagnosis, prevention, and treatment of pertussis. Curr Inf Dis Rep.

[2] Zepp F, Heininger U, Mertsola J, Bernatowska E, Guiso N, Roord J, Tozzi AE, Van Damme P (2011). Rationale for pertussis booster vaccination throughout life in Europe. Lancet Inf Dis.

[3] Kretsinger K, Broder KR, Cortese MM, Joyce MP, Ortega-Sanchez I, Lee GM, Tiwari T, Cohn AC, Slade BA, Iskander JK, Mijalski CM, Brown KH, Murphy TV, Centers for Disease Control and Prevention; Advisory Committee on Immunization Practices; Healthcare Infection Control Practices Advisory Committee (2006). Preventing tetanus, diphtheria, and pertussis among adults: use of tetanus toxoid, reduced diphtheria toxoid and acellular pertussis vaccine recommendations of the Advisory Committee on Immunization Practices (ACIP) and recommendation of ACIP, supported by the Healthcare Infection Control Practices Advisory Committee (HICPAC), for use of Tdap among health-care personnel. MMWR Recomm Rep.

[4] Cherry JD (2012). Epidemic pertussis in 2012 - the resurgence of a vaccine-preventable disease. N Engl J Med.

[5] Rothstein E, Edwards K (2005). Health burden of pertussis in adolescents and adults. Pediatr Inf Dis J.

[6] Bettiol S, Wang K, Thompson MJ, Roberts NW, Perera R, Heneghan CJ, Harnden A (2012). Symptomatic treatment of the cough in whooping cough. Cochrane Database Syst Rev.

[7] Tan T, Trindade E, Skowronski D (2005). Epidemiology of pertussis. Pediatr Inf Dis J.

[8] Mattoo S, Cherry JD (2005). Molecular pathogenesis, epidemiology, and clinical manifestations of respiratory infections due to *Bordetella* pertussis and other *Bordetella* subspecies. Clin Microbiol Rev.

[9] Centers for Disease Control and Prevention (CDC) (2012). Pertussis epidemic - Washington, 2012. MMWR Morbid Mortal Wkly Rep.

[10] Centers for Disease Control and Prevention (CDC) (2012). Updated recommendations for use of tetanus toxoid, reduced diphtheria toxoid, and acellular pertussis (Tdap) vaccine in adults aged 65 years and older - Advisory Committee on Immunization Practices (ACIP). MMWR Morbid Mortal Wkly Rep.

[11] Cherry JD, Tan T, Wirsing von Konig CH, Forsyth KD, Thisyakorn U, Greenberg D, Johnson D, Marchant C, Plotkin S (2012). Clinical definitions of pertussis: Summary of a Global Pertussis Initiative roundtable meeting, February 2011. Clin Infect Dis.

[12] Prince HE, Lieberman JM, Cherry JD (2012). Age-related differences in patterns of increased *Bordetella* pertussis antibodies. Clin Vacc Immunol.

[13] Centers for Disease Control and Prevention (CDC): Pertussis (Whooping Cough): Diagnosis Confirmation (website: http://www.cdc.gov/pertussis/clinical/diagnostic-testing/diagnosis-confirmation.html). Last accessed November 2015.

[14] Cherry JD, Seaton BL (2012). Patterns of *Bordetella* parapertussis respiratory illnesses: 2008–2010. Clin Infect Dis.

[15] Paget WJ, Balderston C, Casas I, Donker G, Edelman L, Fleming D, Larrauri A, Meijer A, Puzelli S, Rizzo C, Simonsen L, EPIA collaborators (2010). Assessing the burden of paediatric influenza in Europe: the European Paediatric Influenza Analysis (EPIA) project. Eur J Pediatr.

[16] Pitman RJ, Melegaro A, Gelb D, Siddiqui MR, Gay NJ, Edmunds WJ (2007). Assessing the burden of influenza and other respiratory infections in England and Wales. J Inf.

[17] Olson DR, Heffernan RT, Paladini M, Konty K, Weiss D, Mostashari F (2007). Monitoring the impact of influenza by age: emergency department fever and respiratory complaint surveillance in New York City. PLoS Med.

[18] McGuiness CB, Hill J, Fonseca E, Hess G, Hitchcock W, Krishnarajah G (2013). The disease burden of pertussis in adults 50 years old and older in the United States: a retrospective study. BMC Inf Dis.

[19] US Census Bureau. Population Estimates. Website: http://factfinder.census.gov/. Last access dates November 2015.

[20] AMA Physician Masterfile In. Website: http://www.ama-assn.org/ama/pub/about-ama/physician-data-resources/physician-masterfile.page. Last access dates November 2015.

[21] Toback SL, Herley J, Edelman L, Ambrose CS (2011). Trends in U.S. pediatric influenza vaccination from 2006 to 2010 among children with private insurance. Vaccine.

[22] Ryan MJ, Ramsay M, Brown D, Gay NJ, Farrington CP, Wall PG (1996). Hospital admissions attributable to rotavirus infection in England and Wales. J Inf Dis.

[23] Muller-Pebody B, Edmunds WJ, Zambon MC, Gay NJ, Crowcroft NS (2002). Contribution of RSV to bronchiolitis and pneumonia-associated hospitalizations in English children, April 1995-March 1998. Epidemiol Inf.

[24] Stolwijk AM, Straatman H, Zielhuis GA (1999). Studying seasonality by using sine and cosine functions in regression analysis. J Epidemiol Commun Health.

[25] Thompson WW, Shay DK, Weintraub E, Brammer L, Cox N, Anderson LJ, Fukuda K (2003). Mortality associated with influenza and respiratory syncytial virus in the United States. JAMA.

[26] Liao CM, Chang SY, Chen SC, Chio CP (2009). Influenza-associated morbidity in subtropical Taiwan. Int J Inf Dis.

[27] Cherry JD (2005). The epidemiology of pertussis: a comparison of the epidemiology of the disease pertussis with the epidemiology of *Bordetella* pertussis infection. Pediatrics.

[28] Ward JI, Cherry JD, Chang SJ, Partridge S, Lee H, Treanor J, Greenberg DP, Keitel W, Barenkamp S, Bernstein DI, Edelman R, Edwards K, APERT Study Group (2005). Efficacy of an acellular pertussis vaccine among adolescents and adults. N Engl J Med.

[29] Hodder SL, Cherry JD, Mortimer EA, Ford AB, Gornbein J, Papp K (2000). Antibody responses to *Bordetella* pertussis antigens and clinical correlations in elderly community residents. Clin Infect Dis.

[30] Centers for Disease Control and Prevention (CDC) (2012). Summary of notifiable diseases - United States, 2010. MMWR Morbid Mortal Wkly Rep.

[31] Nennig ME, Shinefield HR, Edwards KM, Black SB, Fireman BH (1996). Prevalence and incidence of adult pertussis in an urban population. JAMA.

[32] Strebel P, Nordin J, Edwards K, Hunt J, Besser J, Burns S, Amundson G, Baughman A, Wattigney W (2001). Population-based incidence of pertussis among adolescents and adults, Minnesota, 1995–1996. J Inf Dis.

[33] Dragsted DM, Dohn B, Madsen J, Jensen JS (2004). Comparison of culture and PCR for detection of *Bordetella* pertussis and Bordetella parapertussis under routine laboratory conditions. J Med Microbiol.

[34] Dalby T, Fry NK, Krogfelt KA, Jensen JS, He Q (2013). Evaluation of PCR methods for the diagnosis of pertussis by the European surveillance network for vaccine-preventable diseases (EUVAC.NET). Eur J Clin Microbiol Infect Dis.

[35] Centers for Disease Control and Prevention (CDC) (2011). Local health department costs associated with response to a school-based pertussis outbreak - Omaha, Nebraska, September-November 2008. MMWR Morbid Mortal Wkly Rep.

[36] Witt MA, Katz PH, Witt DJ (2012). Unexpectedly limited durability of immunity following acellular pertussis vaccination in preadolescents in a North American outbreak. Clin Infect Dis.

[37] Sotir MJ, Cappozzo DL, Warshauer DM, Schmidt CE, Monson TA, Berg JL, Zastrow JA, Gabor GW, Davis JP (2008). A countywide outbreak of pertussis: initial transmission in a high school weight room with subsequent substantial impact on adolescents and adults. Arch Pediatr Adolesc Med.

[38] Craig AS, Wright SW, Edwards KM, Greene JW, Haynes M, Dake AD, Schaffner W (2007). Outbreak of pertussis on a college campus. Am J Med.

[39] Kretzschmar M, Teunis PF, Pebody RG (2010). Incidence and reproduction numbers of pertussis: estimates from serological and social contact data in five European countries. PLoS Med.

