# Erratum to: The estimated incidence of pertussis in people aged 50 years old or older in the United States, 2006–2010

**DOI:** 10.1186/s12879-016-1518-y

**Published:** 2016-04-25

**Authors:** Cristina Masseria, Girishanthy Krishnarajah

**Affiliations:** Global Health & Value, Pfizer Inc., 235 E 42nd street, New York, NY 10017 USA; Vaccines, GlaxoSmithKline Vaccines, 5 Crescent Dr, Philadelphia, PA 19112 USA

## Erratum

After publication of the original article [1], it came to the authors’ attention that the title had been incorrectly published as **The estimated incidence of pertussis in people aged 50 years old in the United States, 2006–2010**.

“50 years old” should have read as “50 years or older”, so the title should have read: **The estimated incidence of pertussis in people aged 50 years old or older in the United States, 2006–2010**. The correct title of the original article appears in this erratum.
